# Polyunsaturated fatty acid elongation and desaturation in activated human T-cells: ELOVL5 is the key elongase[S]

**DOI:** 10.1194/jlr.M090050

**Published:** 2018-10-06

**Authors:** Philippe-Pierre Robichaud, Jean Eric Munganyiki, Eric Boilard, Marc E. Surette

**Affiliations:** Department of Chemistry and Biochemistry,* Université de Moncton, Moncton, NB, E1A 3E9 Canada; Department of Microbiology, Infectious Diseases and Immunology,† Université Laval, Québec, QC, G1V 4G2 Canada

**Keywords:** metabolism, desaturases, lipids, arachidonic acid, ω-3, elongation of very long chain fatty acids protein 5

## Abstract

PUFAs are important constituents of membrane glycerophospholipids. However, changes in the capacities to incorporate and metabolize PUFAs when cells enter the cell cycle have not been thoroughly studied. In this study, differences in the incorporation and metabolism of exogenous PUFAs in resting and proliferating primary human T-cells and in the Jurkat cell line were measured. Overall, proliferating T-cells and Jurkat cells had a greater capacity to incorporate and elongate exogenous 18- and 20-carbon PUFAs compared with resting T-cells. Proliferating T-cells and Jurkat cells also showed a greater capacity to desaturate 18-carbon PUFA substrates. Consistent with these observations, a significant increase in the expression of fatty acid desaturase (FADS) 1, FADS2, and elongation of very long chain fatty acids protein (ELOVL) 5 was measured in proliferating T-cells compared with resting T-cells. No quantifiable ELOVL2 was measured. Knockdown of ELOVL5 in T-cells and Jurkat cells significantly affected cellular monounsaturated and PUFA profiles and strongly impaired the elongation of 18- and 20-carbon PUFAs. In conclusion, the induction of proliferation in human T-cells is associated with a significant increase in the capacity to take up and metabolize exogenous PUFAs, and ELOVL5 is responsible for the elongation of 18- and 20-carbon PUFAs in these cells.

Cellular proliferation is a natural phenomenon; however, in certain pathologies such as cancers, as well as autoimmune and inflammatory diseases such as rheumatoid arthritis, atherosclerosis, and lupus erythematosus, cell proliferation occurs that contributes to the progression of the pathology. In cancer cells, de novo biosynthesis of saturated fatty acids (SFAs) and MUFAs is enhanced to support glycerophospholipid (GPL) and membrane biosynthesis. This is a result of increased expression of key enzymes such as acetyl-CoA carboxylase, FAS, and stearoyl-CoA desaturase 1 (SCD1) (1–6).

While de novo FA biosynthesis provides many of the substrates for GPL biosynthesis during cell proliferation, GPLs undergo subsequent remodeling via the Lands cycle, which incorporates additional PUFAs into GPLs (7, 8). We previously showed that the expression of many enzymes involved in PUFA-GPL remodeling such as acyl-CoA synthases, lysophospholipid acyl-CoA transferases, and phospholipases A_2_ are modified when cells are stimulated to proliferate (9). Such PUFAs that are not products of de novo FA biosynthesis are important structural components of cellular membranes (10–13) that also perform signaling functions and serve as ligands for nuclear receptors as well as precursors to lipid mediators such as the eicosanoids (14–18). Most PUFAs are members of the n-3 and n-6 families and cannot be synthesized in mammals. These families of PUFAs are obtained through diet, with linoleic acid (LA; 18:2n-6) and α-linolenic acid (ALA; 18:3n-3) being the parent PUFAs in each family. Once incorporated into cells, these 18-carbon PUFAs can undergo a series of elongation and desaturation reactions to generate longer-chain, more unsaturated PUFAs such as arachidonic acid (AA; 20:4n-6), EPA (20:5n-3), and DHA (22:6n-3) (19–23) (**Fig. 1**).

**Fig. 1.**
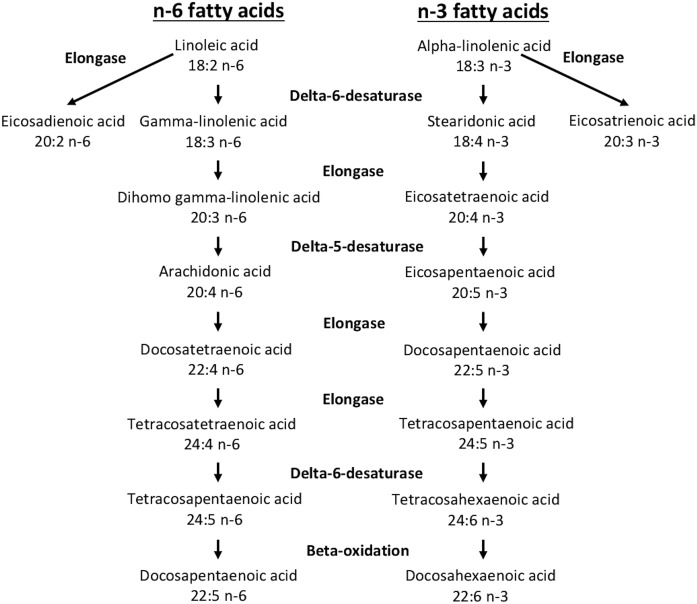
The consensus pathway of the interconversion of PUFAs in mammals (45).

The elongation and desaturation reactions to which PUFAs are subjected within the cell depends on the expression of elongases and desaturases. The cellular PUFAs can be elongated by the elongation of very long chain fatty acids proteins (ELOVLs), of which seven have been identified in humans (19–21, 23), while desaturation reactions are catalyzed by Δ5- and Δ6-desaturases [gene products of fatty acid desaturase (FADS) 1 and FADS2, respectively] (20, 22, 23). However, despite enhanced PUFA-GPL remodeling associated with cell proliferation (9), the differential expression of the enzymes linked to PUFA elongation and desaturation following the induction of cell proliferation is not known. In this study, we report significant changes in the capacity of primary human T-cells to metabolize PUFAs when stimulated to undergo cell proliferation, and these changes are associated with enhanced expression of FADS1, FADS2, and ELOVL5.

## MATERIALS AND METHODS

### Reagents

Human recombinant interleukin-2 (IL-2), boron trifluoride (14% in methanol), 2,3,4,5,6-pentafluorobenzyl bromide, *N*,*N*-diisopropylethylamine, horseradish peroxidase-conjugated anti-β-actin, and the horseradish peroxidase-conjugated anti-rabbit antibodies were from Sigma-Aldrich (Oakville, Canada). The 1,2-diheptadecanoyl-*sn*-glycero-3-phosphocholine was from Biolynx (Brockville, Canada). Fatty acid methyl esters (FAMEs) and FFAs were obtained from Nu-Check Prep (Elysian, MN). Deuterated AA (5,8,11,14-eicosatetraenoic-5,6,8,9,11,12,14,15-d_8_ acid [AA-d_8_]) and deuterated EPA [5,8,11,14,17-eicosapentaenoic-19,19,20,20,20-d_5_ acid (EPA-d_5_)] were from Cayman Chemical (Ann Arbor, MI). Antibodies against acyl-CoA synthetase (ACSL) 4, Δ5-desaturase, Δ6-desaturase, and ELOVL2 were from Abcam (Toronto, Canada). Antibody against ELOVL5 was from OriGene Technologies (Rockville, MD), phycoerythrin-conjugated anti-CD69 was from Biolegend (San Diego, CA), PC7-conjugated anti-CD25 was from Beckman-Coulter (Indianapolis, IN), and the goat anti-rabbit horseradish peroxidase-conjugated secondary antibody was from Jackson ImmunoResearch Laboratories (West Grove, PA). The nonsilencing (NS) negative control siRNA and the siRNA against ELOVL5 were from OriGene Technologies.

### Cell culture

Human peripheral blood mononuclear cells were obtained from blood following centrifugation in Lymphocyte Separation Solution (Wisent Inc., St-Bruno, Canada) as previously described (24). Blood donors were male and female subjects between 18 and 65 years of age who had indicated that they had no known health issues and who had fasted for 12 h prior to the blood draw. T-cells were then isolated by negative selection using the human T-cell enrichment kit from Stem Cell Technologies (Vancouver, Canada) following the manufacturer’s instructions. Primary T-cells and the Jurkat cell line (ATCC, Manassas, VA) were cultured in RPMI-1640 supplemented with 10% FBS, 100 U/ml penicillin, 10 µg/ml streptomycin, 10 mM HEPES, d-glucose (up to 25 mM), and 1 mM sodium pyruvate at 37°C in a 5% CO_2_ atmosphere. Jurkat cells are a human leukemic T-cell lymphoblast cell line that is often used to study T-cell functions. T-cells were stimulated with anti-CD3/anti-CD28 Dynabeads (1 × 10^6^ beads/ml) (Invitrogen) according to the manufacturer’s instructions in the presence of 30 U/ml IL-2 (Sigma-Aldrich) for up to 72 h before all experiments. HepG2 cells (ATCC) were cultured in Eagle’s minimal essential medium supplemented with 10% FBS, 100 U/ml penicillin, and 10 µg/ml streptomycin at 37°C in a 5% CO_2_ atmosphere.

### Gene expression analysis

Total mRNA was extracted from resting and stimulated T-cells using Ribozol reagent (AMRESCO), and the extracted mRNA was purified with the Direct-zol kit (Zymo Research). mRNA was quantified by absorbance at 260 nm, and its purity was evaluated by the ratios of absorbance at 260/280 nm (∼2.0) and 260/230 nm (2.0–2.2). RNA integrity was also evaluated by electrophoretic migration on a 1% agarose gel. mRNA reverse transcription was performed on 1 µg RNA using the QuantiTect Reverse Transcription Kit (QIAGEN). Gene expression was evaluated by quantitative PCR (ABI 7500; Applied Biosystems) using PrimeTime assays (Integrated DNA Technologies) with PerfeCTa qPCR SuperMix Low ROX (Quanta Biosciences). The efficiency of primer pairs (**Table 1**) was evaluated using a standard curve, and the stability of the RN18S1 reference gene expression between treatments (Ct < 1) was verified. This confirms previous reports of RN18S1 as an appropriate reference gene in stimulated human T-cells (25). Specific primers for ELOVL2, ELOVL5, FADS1, FADS2, and RN18S1 were created by using PrimerQuest (Integrated DNA Technologies).

**TABLE 1.** List of primer sequences used in quantitative PCR experiments and product size for each of the indicated transcripts

Transcript (Accession)	Primers	Sequences	Products (bp)
ELOVL2	Forward	TTGGAATCACACTTCTCTCCGCGT	141
NM_017770	Probe	56-FAM/TCCACTTGG/ZEN/GAAGGAGGCTACAACTT/3IABkFQ	
	Reverse	AGTACCACCAAAGCACCTTGGCTA	
ELOVL2	Forward	TGTGTCCAGGAACTCTACTGA	111
NM_017770	Probe	56-FAM/TTGGCTACC/ZEN/CGGATGTCAGCTTC/3IABkFQ	
	Reverse	GGCTACAACTTACAGTGTCAAGA	
ELOVL5	Forward	TTCATCCTGCGCAAGAACAACCAC	188
NM_021814	Probe	56-FAM/TACCACCAT/ZEN/GCCTCGATGCTGAACAT/3IABkFQ	
	Reverse	ATGGAAGGGACTGACGACAAACCA	
FADS1	Forward	AAGCAACTGGTTTGTGTGGGTGAC	198
NM_013402	Probe	56-FAM/AGGCCACAT/ZEN/GCAATGTCCACAAGTCT/3IABkFQ	
	Reverse	TAATTGTGTCGAGGCATCGTGGGA	
FADS2	Forward	TACGCTGGAGAAGATGCAACGGAT	139
NM_004265	Probe	56-FAM/TGACCTGGA/ZEN/ATTCGTGGGCAAGTTCT/3IABkFQ	
	Reverse	TCTTTGAGTTCTTGCCGTGGTCCT	
RN18S1	Forward	GAGACTCTGGCATGCTAACTAG	129
NR_003286	Probe	56-FAM/TGCTCAATC/ZEN/TCGGGTGGCTGAA/3IABkFQ	
	Reverse	GGACATCTAAGGGCATCACAG	

### Western blot analysis

Cells were washed twice with PBS, and lysis buffer (150 mM NaCl; 1% Nonidet P-40; 2 mM EDTA; and 50 mM Tris-HCL, pH 7.6) containing protease inhibitor cocktail (Roche) was added to the pellets. After complete homogenization, proteins were quantified by the Microplate BCA Protein Assay Kit (Pierce). 5× Laemmli sample buffer was added, and samples were heated 10 min at 40°C (9). Cellular proteins (10 μg) were separated on Criterion 4–15% polyacrylamide gels (Bio-Rad) and transferred onto a PVDF membrane. Membranes were incubated with indicated primary antibodies and horseradish peroxidase-conjugated secondary antibodies. Western blots were developed using Amersham ECL Prime (GE Healthcare), and images were captured using an Alpha Innotech FluorChem imager.

### Incubation with FAs

After 3 days of incubation for primary T-cells or 2 days of incubation for Jurkat and HepG2 cells since the last split, the incorporation and metabolism of PUFAs were evaluated by incubating resting T-cells, stimulated T-cells, Jurkat cells, or HepG2 cells for 24 h with different PUFAs or their diluent (0.05% ethanol) in the cells’ respective culture media containing 10% FBS as described above. Stimulated T-cells, Jurkat cells, and HepG2 cells were incubated with 5 µM of each PUFA, whereas resting T-cells were incubated with 15 µM of each PUFA. The PUFAs utilized were LA, γ-linolenic acid (GLA; 18:3n-6), AA, ALA, stearidonic acid (SDA; 18:4n-3), EPA, AA-d_8_, and EPA-d_5_.

### Lipid extraction and quantification of FAs by GC

After 24 h of incubation with different PUFAs or their diluent, cells were washed by centrifugation using PBS containing 1 mg/ml BSA, and cellular lipids were extracted into chloroform containing 3.2 μg of the internal standard 1,2-diheptadecanoyl-*sn*-glycerol-3-phosphorylcholine (Biolynx) using the Bligh and Dyer method (26). The extracted lipids were saponified with 0.5 M KOH in methanol (100°C for 15 min), and FAMEs were prepared by adding 14% BF_3_ in methanol (100°C for 15 min) and quantified by GC with flame ionization detection (FID) as previously described (9, 27). FAME standards (Nu-Check Prep) were used to identify peak retention times, and standard curves were used for FAME quantification. For the deuterated FA experiments, pentafluorobenzyl esters of FAs were prepared and measured by negative ion chemical ionization GC/MS using a Polaris Q mass spectrometer (Thermo Electron Corporation) as previously described (9, 27).

### Gene knockdown

Jurkat cells, proliferating T-cells, and HepG2 cells cultured without penicillin and streptomycin for at least 24 h before electroporation were washed twice with RPMI without l-glutamine. Electroporation (270 V and 600 μF) was done in 0.4 cm cuvettes on 1 × 10^7^ cells in 300 μl RPMI without l-glutamine in the presence of 300 nM NS siRNA or siRNA against ELOVL5. Cells were transferred to culture flasks prewarmed with complete culture medium without penicillin and streptomycin. Cells were verified for viability after 48 h by trypan blue exclusion under a light microscope.

### Cell proliferation assays

Cell proliferation was measured by flow cytometry using the CellTrace CFSE Cell Proliferation Kit (Thermo Fisher Scientific). Briefly, wild-type Jurkat cells were stained with 1 μM CFSE in PBS for 20 min, and unincorporated CFSE was quenched and washed with complete culture medium following the manufacturer’s protocol. Stained cells were incubated for 48 h before their transfection with the NS control siRNA and the siRNA against the ELOVL5. Cells were then analyzed by flow cytometry (FC500; Beckman-Coulter) at 48 and 96 h posttransfection.

Cell proliferation was also measured using the Click-iT EdU Alexa Fluor 488 Flow Cytometry Assay Kit (Thermo Fisher Scientific) in combination with the FxCycle Violet Stain (Thermo Fisher Scientific) following the manufacturer’s protocol. Briefly, at 72 and 96 h posttransfection, Jurkat cells were incubated with 10 µM 5-ethynyl-2′-deoxyuridine (EdU) for 2 h at 37°C, washed, fixed, and permeabilized, and the Click-iT reaction was done to conjugate the incorporated EdU molecules to the Alexa Fluor 488. All components used were provided with the kit. Cells were then resuspended in 1 ml PBS and stained with the FxCycle Violet Stain following the manufacturer’s protocol before being analyzed by flow cytometry (FC500; Beckman-Coulter).

### Apoptosis assay

Jurkat cells transfected with the NS control siRNA and the siRNA against the ELOVL5 and incubated for 72 h were stained with annexin V (BioLegend) and with propidium iodide (Invitrogen) following the manufacturer’s protocol and analyzed by flow cytometry (FC500; Beckman-Coulter).

### Markers of T-cell activation

T-cells were stimulated with anti-CD3/anti-CD28 as described above with or without the addition of IL-2. After 18 h, cells were transfected with NS siRNA or the siRNA against the ELOVL5, and incubation was continued for an additional 48 h as described above. Expression of the T-cell activation markers CD25 and CD69 was then measured by flow cytometry (FC500; Beckman-Coulter).

### Statistics

GraphPad Prism version 6.0 was used to perform the statistical analyses.

### Ethics

This study was approved by the Université de Moncton Institutional Review Committee for Research involving human subjects (approval number 1314-029). All subjects provided written informed consent prior to their participation in the study.

## RESULTS

### Primary T-cell culture and proliferation

After 3 days of incubation, the stimulated T-cells grew in clusters, and the cell size and cell counts were increased compared with resting cells (mean ± SEM: 2.4 ± 0.2-fold; *P* < 0.0001 as determined by Student’s *t*-test; *n* = 8), in accordance with previous reports (9, 28–30).

### Supplementation with PUFAs in T-cells and Jurkat cells

In preliminary experiments, cells were incubated with 5 µM exogenous PUFAs for 24 h. However, resting T-cells incorporated very little FAs, and thus PUFA metabolism was difficult to assess. Therefore, all further experiments with resting T-cells utilized PUFA concentrations of 15 µM. This difference in the capacity of resting and proliferating T-cells to take up exogenous AA is consistent with previous reports of a significantly enhanced capacity to incorporate [^3^H]AA in stimulated T-cells in pulse-labeling experiments (9).

### Incorporation and metabolism of n-6 PUFAs

**Fig. 2.**
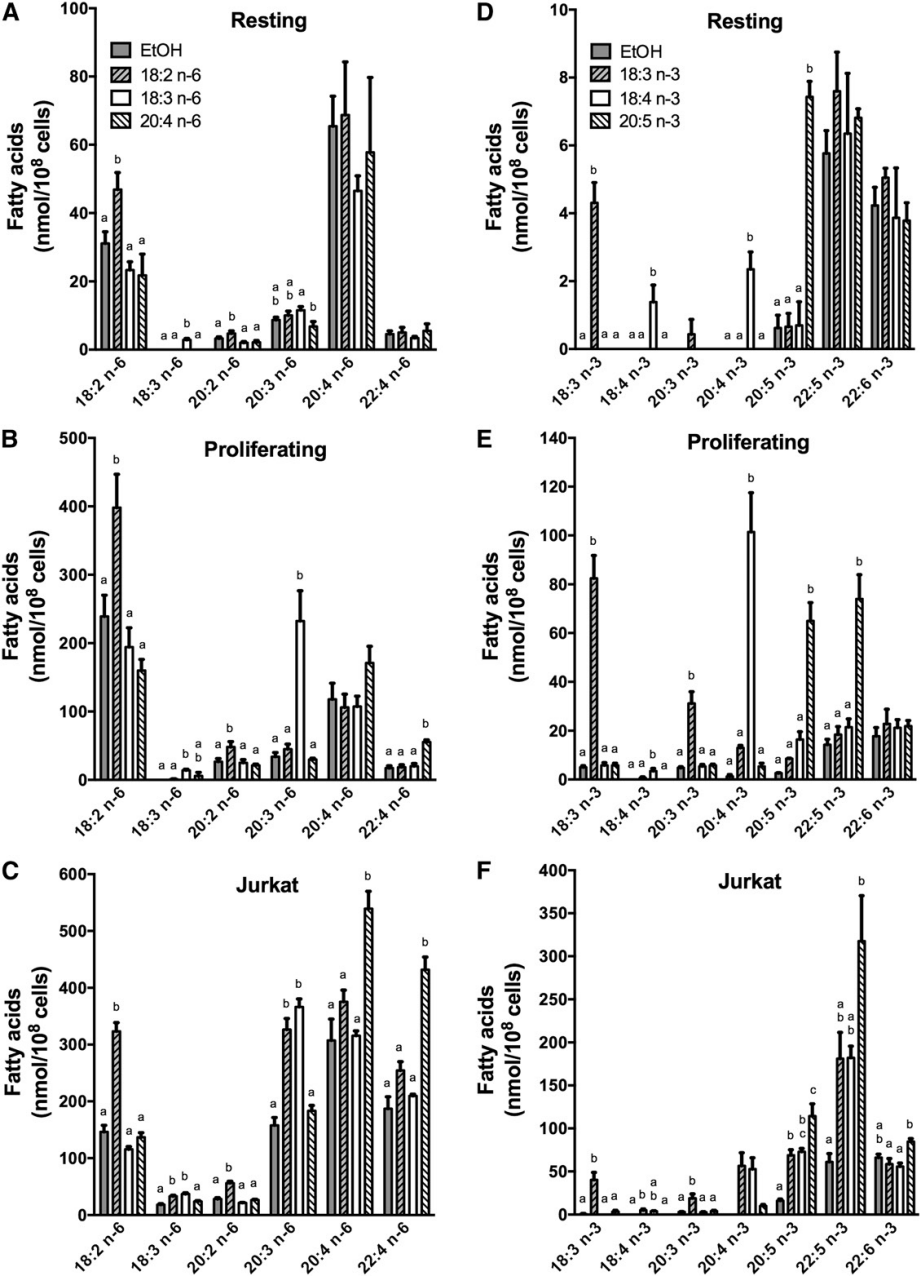
The mass content of n-6 and n-3 FAs in resting T-cells, proliferating T-cells, and Jurkat cells following supplementation with different PUFAs. Resting T-cells were incubated without stimulation, and proliferating T-cells were incubated with anti-CD3/anti-CD28 beads in the presence of 30 U/ml IL-2 for 3 days. T-cells and Jurkat cells were then incubated for 24 h with different PUFAs (18:2n-6, 18:3n-6, 20:4n-6, 18:3n-3, 18:4n-3, or 20:5 n-3) or ethanol as the control. Resting T-cells (A, D) were incubated with 15 µM of each FA, whereas proliferating T-cells (B, E) and Jurkat cells (C, F) were incubated with 5 µM of each PUFA. Cellular lipids were extracted, hydrolyzed, and transmethylated. Individual FAs were measured by GC-FID. The results are means ± SEMs of three (with n-3 PUFAs) or four (with n-6 PUFAs) independent experiments. Each independent experiment was conducted with cells obtained from a different subject. Cells were obtained from two males and two females. Values for each measured FA that do not have a common superscript are significantly different (*P* < 0.05) as determined by one-way ANOVA with repeated measures and Tukey’s post hoc test. EtOH, ethanol.

When cells were incubated with 18:2n-6 (LA), there was a significant increase in the cellular content of LA and of its elongation product 20:2n-6 in resting T-cells compared with nonsupplemented controls (**Fig. 2A**). The accumulation of LA compared with nonsupplemented controls that was measured in proliferating T-cells and in Jurkat cells was also accompanied by an augmentation of cellular 20:2n-6 content; however, in Jurkat cells there was also an increase in 18:3n-6 and 20:3n-6 (Fig. 2B, C).When cells were incubated with 18:3n-6 (GLA), only the accumulation of a small quantity of GLA was measured in resting T-cells that was different from controls (Fig. 2A). In proliferating T-cells a small increase in cellular GLA was also measured; however, a significant accumulation of its elongation product 20:3n-6 was measured, indicating that T-cell stimulation enhanced the cells’ capacity to incorporate and elongate GLA (Fig. 2B). In Jurkat cells there was also a large increase of 20:3n-6 content compared with controls (Fig. 2C). When cells were incubated with 20:4n-6 (AA), there was no change in the n-6 PUFA content of resting T-cells compared with controls, while in proliferating T-cells and Jurkat cells a significant increase in both AA and 22:4n-6 content was measured (Fig. 2A–C).

Overall, these results indicate that T-cell stimulation increases the capacity of the cells to take up and elongate these PUFAs. Indeed, these molar data demonstrate the much greater capacity of stimulated T-cells and Jurkat cells to take up exogenous FAs after a 24 h incubation based on the increase from baseline in cellular PUFA content (>100 nmol/10^8^ cells) compared with resting T-cells (<20 nmol/10^8^ cells) despite the resting cells having been exposed to greater concentrations of exogenous PUFAs. Importantly, the incubation of the cells with these FAs did not have an impact on the total FA pool, as the total mass of FAs per cell was not significantly changed (supplemental Table S1, supplemental Table S2). This suggests that the PUFA concentrations of 15 µM for resting cells and of 5 µM for proliferating cells did not cause an imbalance in overall cellular FA content. These patterns of changes were similar when comparisons of the percentage of total FAs were made (supplemental Fig. S1A–C).

### Incorporation and metabolism of n-3 PUFAs

When cells were incubated with 18:3n-3 (ALA), a significant increase in cellular ALA was measured in resting and proliferating T-cells compared with controls (Fig. 2D–E). Unlike resting cells, a significant increase in cellular 20:3n-3 content was also measured in proliferating T-cells, indicating a greater capacity to elongate n-3 PUFA than in resting T-cells. In Jurkat cells incubated with ALA, significant increases in ALA, 18:4n-3, 20:3n-3, EPA, and 22:5n-3 were measured compared with controls. The extent of the metabolism of ALA to 22:5n-3 indicates that Jurkat cells have a greater capacity to elongate and desaturate PUFAs than primary T-cells (Fig. 2F). When cells were incubated with 18:4n-3 (SDA), a small accumulation of SDA and an increase in the cellular content of its elongation product 20:4n-3 was measured in resting T-cells compared with controls. The elongation of incorporated SDA to 20:4n-3 was significantly more pronounced in proliferating T-cells (an increase in 20:4n-3 of 2.4 ± 0.5 nmol/10^8^ cells in resting cells and 100.2 ± 16.6 nmol/10^8^ cells in proliferating cells; *P* < 0.05 as determined by paired Student’s *t*-test). In Jurkat cells, a significant increase in EPA was measured (Fig. 2D–F). When cells were incubated with 20:5n-3 (EPA), there was an accumulation of EPA in all cells, and a significant increase in the cellular 22:5n-3 content was also measured in proliferating T-cells and Jurkat cells compared with nonsupplemented controls (Fig. 2D–F). As with n-6 PUFAs, these molar mass data show the much greater capacity of stimulated T-cells and Jurkat cells to take up exogenous FAs compared with resting T-cells. These patterns of change were similar to those observed when comparisons of the percentage of total FAs were made (supplemental Fig. S1D–F).

Overall, these results show that the stimulation of primary T-cells to proliferate is associated with an enhanced capacity to elongate 18-carbon PUFAs, a newly acquired capacity to elongate 20-carbon PUFAs, and a newly acquired capacity to desaturate 18- and 20-carbon PUFAs. The Jurkat cell line showed a capacity to metabolize PUFAs through all desaturation and elongation steps compared with primary T-cells.

### Gene expression analyses

Given the significant increase in the capacities for cellular PUFA metabolism when peripheral T-cells are stimulated to proliferate, the expression of potentially related genes were measured by quantitative PCR (**Table 2**). The significant increase in the capacity to elongate PUFAs in proliferating T-cells compared with resting T-cells was associated with a significant increase in the expression of ELOVL5. However, no signal for ELOVL2, another elongase known to prefer long-chain PUFAs as substrates, was measured in T-cells despite the design of two different primer pairs (see Table 1). In addition, the expression of FADS1 and FADS2 was also significantly increased in proliferating T-cells compared with resting T-cells, again consistent with the enhanced capacity to desaturate exogenously provided PUFAs in proliferating T-cells.

**TABLE 2.** Gene expression of selected enzymes in resting and proliferating T-cells

Genes	Fold Increase
ELOVL2	ND
ELOVL5	1.6 ± 0.2*
FADS1 (Δ-5)	16.4 ± 1.7*
FADS2 (Δ-6)	10.3 ± 0.6*

RNA from resting and proliferating T-cells was extracted, purified, and reverse-transcribed into cDNA. Quantitative PCR was performed using RN18S1 as the reference gene. Values represent the means ± SEMs of fold increases of RNA expression in proliferating T-cells compared with resting T-cells for three to six independent experiments. Each independent experiment was conducted with cells obtained from a different subject. *Different from resting cells (*P* < 0.05) as determined by paired two-sided Student’s *t*-test. ND, not detected.

### Protein expression analysis

**Fig. 3.**
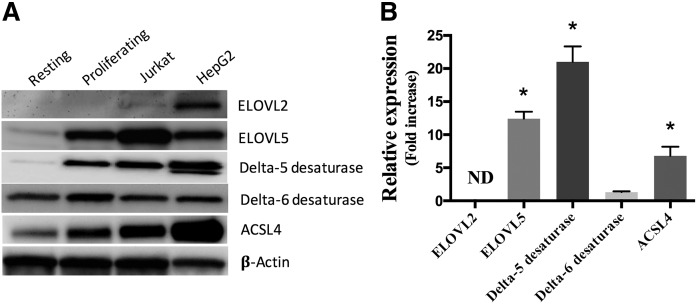
Protein expression of indicated enzymes. Proteins (10 μg) from primary resting and proliferating T-cells, Jurkat cells, and HepG2 cells were separated on 10% SDS-PAGE gels and transferred onto a PVDF membrane. Western blotting was performed using primary and secondary antibodies as listed in the Material and Methods section. A: Western blots representative of three independent experiments. B: The relative expression of proteins in proliferating T-cells compared with resting T-cells was determined by densitometry using β-actin for normalization. The values are means ± SEMs of three independent experiments. For T-cells, each independent experiment was conducted with cells obtained from a different subject. *Different from resting cells (*P* < 0.05) as determined by paired two-sided Student’s *t*-test. ND, not determined.

Protein expression of elongases (ELOVLs), desaturases, and ACSL4 known to utilize PUFAs were measured (**Fig. 3**). The protein expression of Δ5-desaturase as well as that of ELOVL5 was induced in proliferating T-cells compared with resting T-cells. However, unlike gene expression results, Δ6-desaturase expression was not significantly increased. No quantifiable ELOVL2 protein expression was measured in primary human T-cells or in the Jurkat cell line. As a positive control, both ELOVL2 and ELOVL5 proteins were expressed in hepatic HepG2 cells. Because ACSLs are implicated in cellular FA uptake and are required for the elongation and desaturation of fatty acyl-CoA, the protein expression of ACSL4 known to utilize PUFAs as substrates was measured by Western blot and was shown to be induced in stimulated proliferating T-cells compared with resting cells (Fig. 3).

### ELOVL5 silencing in Jurkat cells

**Fig. 4.**
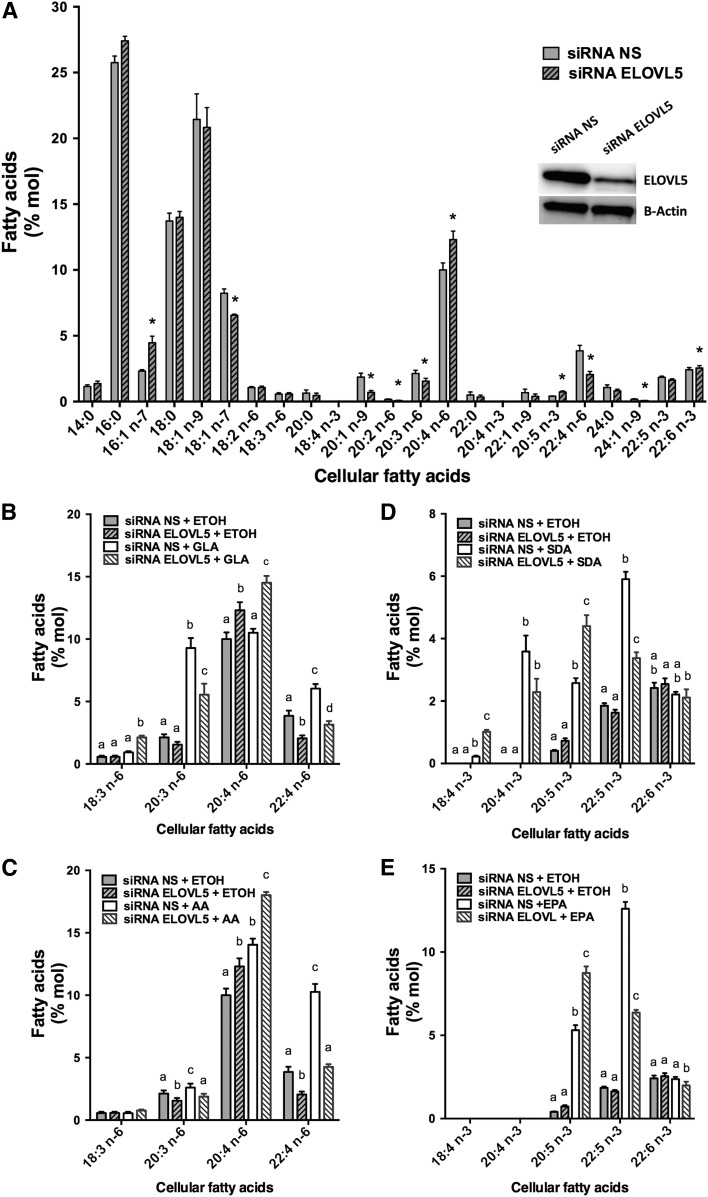
Jurkat cell FA distribution following ELOVL5 knockdown and supplementations with or without different PUFAs. Jurkat cells were transfected with NS control siRNA and siRNA against ELOVL5. Cells were incubated for 48 h in regular culture media before a 24 h supplementation with 5 µM of different PUFAs (18:2n-6, 18:3n-6, 20:4n-6, 18:3n-3, 18:4n-3, or 20:5n-3) or ethanol as the control. A: FA composition of cells transfected with the NS siRNA and the siRNA against the ELOVL5. The insert is the Western blot against ELOVL5 and β-actin on protein from cells transfected with the NS siRNA and the siRNA against the ELOVL5. *Different from cells transfected with the NS siRNA (*P* < 0.05) as determined by paired two-sided Student’s *t*-test. B: n-6 PUFA composition of controls and cells incubated with GLA (18:3n-6). C: n-6 PUFA composition of controls and cells incubated with AA (20:4n-6). D: n-3 PUFA composition of controls and cells incubated with SDA (18:4n-3). E: n-3 PUFA composition of controls and cells incubated with EPA (20:5n-3). Cellular lipids were extracted, hydrolyzed, and transmethylated. Individual FAs were measured by GC-FID. The results are means ± SEMs of four independent experiments. Different values for each PUFA that do not have a common superscript are significantly different (*P* < 0.05) as determined by one-way ANOVA with repeated measures and Tukey’s post hoc test. ETOH, ethanol.

The elongases ELOVL2 and ELOVL5 are the enzymes known to prefer 18- and 20-carbon n-3 and n-6 PUFAs (19–21, 23). Because primary T-cells and Jurkat cells did not express measurable ELOVL2, silencing experiments targeting ELOVL5 were performed in proliferating T-cells and in Jurkat cells to measure the impact on PUFA metabolism. Cell viability was greater than 90% 48 h after transfection, as assessed by trypan blue exclusion. The silencing of ELOVL5 in Jurkat cells, which was confirmed by Western blot with a 69 ± 1.3% decrease in expression (*P* < 0.0001 as determined by paired Student’s *t*-test; *n* = 4), caused a significant increase in cellular AA and EPA that was associated with a decrease in cellular 22:4n-6 (**Fig. 4A**).

The role of ELOVL5 in the elongation of 18- and 20-carbon n-3 and n-6 PUFAs was more evident following the supplementation of the culture media of transfected cells with GLA, AA, SDA, and EPA (Fig. 4B–E). In almost all supplementations, silencing of ELOVL5 was associated with an accumulation of the added substrate and a nearly complete loss of the elongation of the supplemented FA. For example, when Jurkat cells were transfected with the NS siRNA and supplemented with AA, a significant accumulation of AA was measured, and the amount of its elongation product, 22:4n-6, doubled (Fig. 4C). However, silencing of ELOVL5 was associated with a greater accumulation of AA, and the amount of its elongation product, 22:4n-6, was comparable to that of nonsupplemented cells (Fig. 4C).

In addition, an increase in 16:1n-7 coupled to a decrease in 18:1n-7 in ELOVL5 silencing experiments suggested that ELOVL5 is not only implicated in the elongation of PUFAs but also of n-7 MUFAs. Moreover, the cellular content of the longer-chain n-9 MUFAs, 20:1n-9 and 24:1n-9, was significantly decreased when ELOVL5 was silenced compared with controls (Fig. 4A).

### ELOVL5 silencing in T-cells

**Fig. 5.**
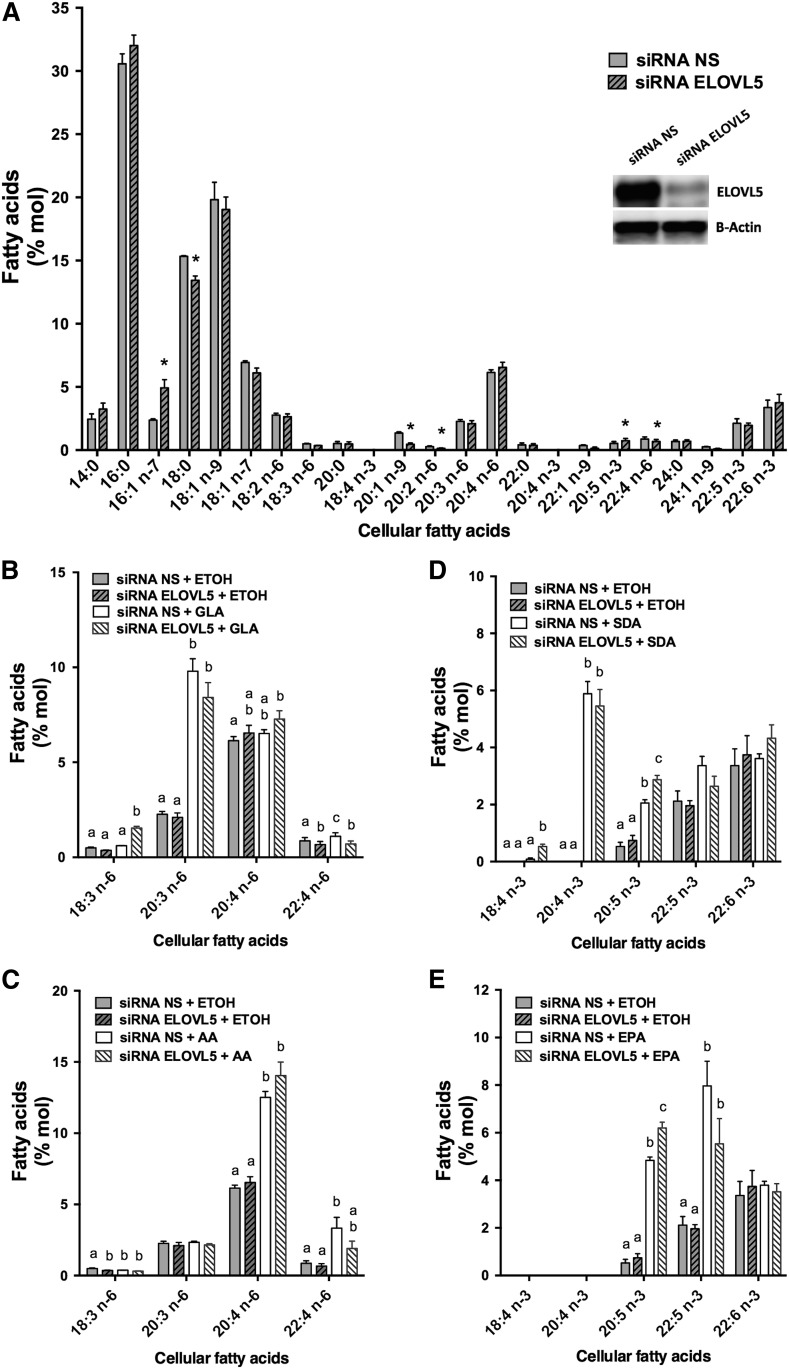
Primary proliferating T-cell FA distribution following ELOVL5 knockdown with and without supplementation with different PUFAs. Proliferating T-cells (48 h after stimulation) were transfected with NS control siRNA and siRNA against ELOVL5. Cells were incubated for 48 h in regular culture media with stimulation before a 24 h supplementation with 5 µM of different PUFAs (18:2n-6, 18:3n-6, 20:4n-6, 18:3n-3, 18:4n-3, or 20:5n-3) or ethanol as the control. A: FA composition of cells transfected with the NS siRNA and the siRNA against the ELOVL5. The insert is the Western blot against ELOVL5 and β-actin on protein from cells transfected with the NS siRNA and the siRNA against the ELOVL5. *Different from cells transfected with the NS siRNA (*P* < 0.05) as determined by paired two-sided Student’s *t*-test. B: n-6 PUFA composition of controls and cells incubated with GLA (18:3n-6). C: n-6 PUFA composition of controls and cells incubated with AA (20:4n-6). D: n-3 PUFA composition of controls and cells incubated with SDA (18:4n-3). E: n-3 PUFA composition of controls and cells incubated with EPA (20:5n-3). Cellular lipids were extracted, hydrolyzed, and transmethylated. Individual FAs were measured by GC-FID. The results are means ± SEMs of three independent experiments. Each independent experiment was conducted with cells obtained from a different subject. Different values for each PUFA that do not have a common superscript are significantly different (*P* < 0.05) as determined by one-way ANOVA with repeated measures and Tukey’s post hoc test. ETOH, ethanol.

The silencing of ELOVL5 in proliferating T-cells (70 ± 4.0% decrease; *P* = 0.003 as determined by paired Student’s *t*-test; *n* = 3) also affected SFAs and MUFAs (**Fig. 5A**), but the impact on the cellular PUFA profile was not as evident as with Jurkat cells.

In PUFA supplementation experiments, silencing of ELOVL5 caused an accumulation of the added substrate in some experiments, and in others it prevented the elongation of the supplemented PUFAs (Fig. 5B–E). For example, when proliferating T-cells were transfected with the NS siRNA and supplemented with GLA, a significant accumulation of its elongation product, 20:3n-6, was observed. This accumulation of 20:3n-6 was not significantly decreased in GLA-supplemented cells when ELOVL5 was silenced, but a significant accumulation of the supplemented FA (GLA) was measured (Fig. 5B). In another example, when proliferating T-cells were transfected with the NS siRNA and supplemented with AA, a significant accumulation of AA and its elongation product, 22:4n-6, was observed. The accumulation of AA was not enhanced in AA-supplemented cells when ELOVL5 was silenced, but the amount of its elongation product (22:4n-6) was no longer significantly increased (Fig. 5C).

### ELOVL5 silencing in HepG2 hepatocarcinoma cells

To compare the observations in T-cells and Jurkat cells with a nonlymphoid cell type, ELOVL5 was silenced in the HepG2 hepatocarcinoma cell line (82 ± 5.2% decrease; *P* = 0.004 as determined by paired Student’s *t*-test; *n* = 3), which is frequently used to study ELOVL proteins and which also expresses ELOVL2 (Fig. 3).

**Fig. 6.**
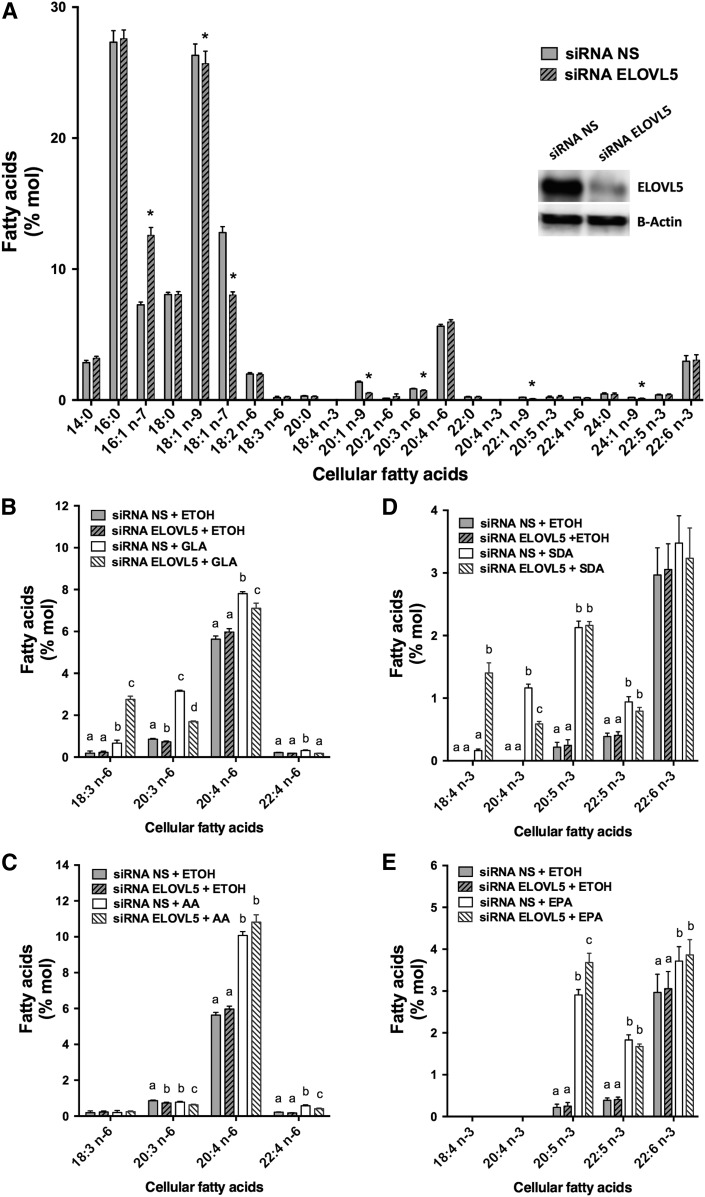
HepG2 cell FA distribution following ELOVL5 knockdown with and without supplementation with different PUFAs. HepG2 cells were transfected with NS control siRNA and siRNA against ELOVL5. Cells were incubated for 48 h in regular culture media before a 24 h supplementation with 5 µM of different PUFAs (18:2n-6, 18:3n-6, 20:4n-6, 18:3n-3, 18:4n-3, or 20:5n-3) or ethanol as the control. A: FA composition of cells transfected with the siRNA NS and the siRNA against the ELOVL5. The insert is the Western blot against ELOVL5 and β-actin on protein from cells transfected with the NS siRNA and the siRNA against the ELOVL5. *Different from cells transfected with the NS siRNA (*P* < 0.05) as determined by paired two-sided Student’s *t*-test. B: n-6 PUFA composition of controls and cells incubated with GLA (18:3n-6). C: n-6 PUFA composition of controls and cells incubated with AA (20:4n-6). D: n-3 PUFA composition of controls and cells incubated with SDA (18:4n-3). E: n-3 PUFA composition of controls and cells incubated with EPA (20:5n-3). Cellular lipids were extracted, hydrolyzed, and transmethylated. Individual FAs were measured by GC-FID. The results are means ± SEMs of four independent experiments. Different values for each PUFA that do not have a common superscript are significantly different (*P* < 0.05) as determined by one-way ANOVA with repeated measures and Tukey’s post hoc test. ETOH, ethanol.

Although these cells are not as rich in cellular PUFAs, modifications in the cellular content of MUFAs and PUFAs were also observed following ELOVL5 silencing (**Fig. 6A**). Notably, ELOVL5 silencing again resulted in an accumulation of 16:1n-7 and a decrease in 18:1n-7, as well as significant decreases in 20-, 22- and 24-carbon n-9 MUFA content. In PUFA supplementation experiments, ELOVL5 silencing affected the capacity to elongate 18-carbon PUFAs with little measurable impact on the elongation of 20-carbon PUFAs (Fig. 6B–E). For example, when HepG2 cells were transfected with the siRNA against the ELOVL5 and supplemented with GLA, the accumulation of its elongation product, 20:3n-6, was significantly reduced, and GLA was significantly increased compared with NS controls supplemented with GLA (Fig. 6B). A similar effect was observed in cells incubated with 18:4n-3.

### Metabolism of deuterated PUFAs

**Fig. 7.**
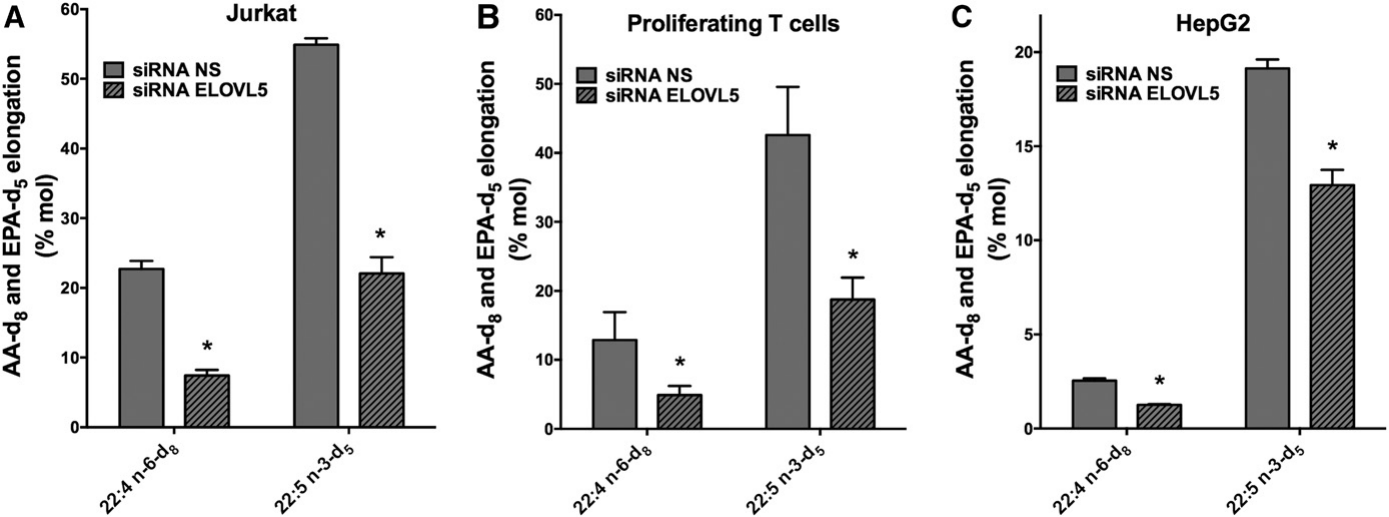
The elongation of AA-d_8_ and EPA-d_5_ following ELOVL5 knockdown in Jurkat cells, proliferating primary T-cells, and HepG2 cells. Jurkat cells (A), proliferating T-cells (B), and HepG2 cells (C) were transfected with NS control siRNA and siRNA against ELOVL5. Cells were incubated for 48 h in regular culture media before a 24 h supplementation with 5 µM AA-d_8_ or EPA-d_5_. Cellular lipids were extracted and hydrolyzed, and pentafluorobenzyl esters of FAs were prepared and measured by negative ion chemical ionization GC/MS. The values represent the percentage of the cellular AA-d_8_ and EPA-d_5_ that was elongated to 22:4-d_8_ and 22:5-d_5_, respectively. The results are means ± SEMs of three independent experiments. *Different from cells transfected with the NS siRNA (*P* < 0.05) as determined by paired two-sided Student’s *t*-test.

To more directly evaluate the elongation of exogenously added AA and EPA, deuterated FAs (AA-d_8_ and EPA-d_5_) were used for the cell supplementation experiments, and GC/MS was used to measure their incorporation into cells as well as their elongation products 22:4n-6-d_8_ and 22:5n-3-d_5_ (**Fig. 7A**–C).

When Jurkat cells transfected with the NS siRNA were supplemented with AA-d_8_ and EPA-d_5_ for 24 h, approximately 23% of the incorporated AA-d_8_ was elongated to 22:4n-6-d_8_, and 55% of the incorporated EPA-d_5_ was elongated to 22:5n-3-d_5_. However, in Jurkat cells transfected with the siRNA against ELOVL5 only 7% of the incorporated AA-d_8_ was elongated to 22:4n-6-d_8_ (70% decrease in elongation), and only 22% of the incorporated EPA-d_5_ was elongated to 22:5n-3-d_5_ (60% decrease in elongation) (Fig. 7A). The ELOVL5 knockdown in primary T-cells also significantly decreased the elongation of AA-d_8_ to 22:4n-6-d_8_ from 13% to 4% (70% decrease) and the elongation of EPA-d_5_ to 22:5n-3-d_5_ from 43% to 19% (56% decrease) (Fig. 7B). In HepG2 cells, the knockdown of ELOVL5 significantly decreased the elongation of AA-d_8_ to 22:4n-6-d_8_ from 3% to 1.3% (57% decrease) and significantly decreased the elongation of EPA-d_5_ to 22:5n-3-d_5_ from 19% to 13% (32% decrease) (Fig. 7C). Of note, in all cell types the efficiency of the elongation of EPA was greater than that of AA, but the elongation of AA was more affected than that of EPA in ELOVL5 knockdown experiments.

### ELOVL5 silencing, apoptosis, cell proliferation, and T-cell activation markers

**Fig. 8.**
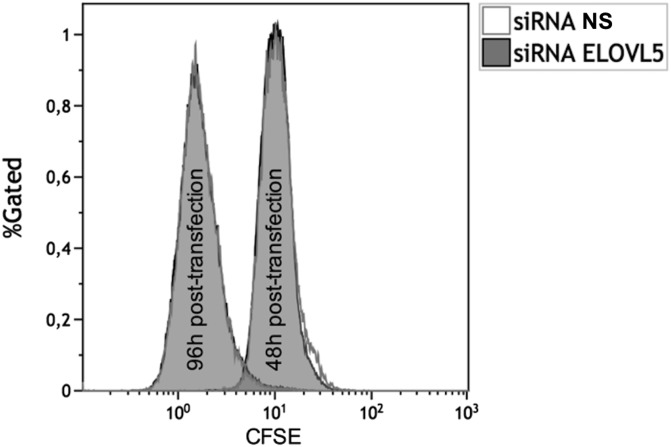
Proliferation of Jurkat cells measured by the CellTrace CFSE flow cytometry cell proliferation assay following ELOVL5 knockdown. Jurkat cells were stained with 1 μM CFSE in PBS for 20 min following the manufacturer’s protocol. Stained cells were then incubated for 48 h before their transfection with the NS control siRNA and the siRNA against the ELOVL5. Cells were then analyzed by flow cytometry at 48 and 96 h posttransfection. The tracings for the siRNA against the ELOVL5 cells are superimposed on those of the NS siRNA cells. These results are representative of three independent experiments.

The impact of ELOVL5 knockdown on cell proliferation and cell death was evaluated in Jurkat cells. ELOVL5 knockdown had no effect on Jurkat cell proliferation as assessed by the rate of CFSE distribution to daughter cells for up to 96 h, as shown in the fluorescence cytograms of cells treated with negative control NS siRNA and siRNA against ELOVL5, which were superimposed at all time points (**Fig. 8**). Similarly, there were no significant differences over 96 h in the staining of cells with the thymidine analogue EdU and propidium iodide analogue FxCycle, indicating that the cell cycle was not affected by ELOVL5 knockdown (supplemental Fig. S2). Apoptosis was also measured in cells treated with NS siRNA and siRNA against ELOVL5, and there was no significant difference in annexin V/propidium iodide staining between the two treatments (supplemental Fig. S3). In addition, the expression of the T-cell activation markers, CD25 and CD69, was not affected by ELOVL5 knockdown in stimulated primary T-cells (supplemental Fig. S4). Overall, these results indicate that ELOVL5 expression does not appear to be required for the maintenance of cell proliferation and viability or the activation of primary T-cells under these culture conditions.

## DISCUSSION

In many cancers both de novo SFA biosynthesis and SCD1 (Δ9-desaturase) expression are significantly increased and thus likely to generate a supply of SFAs and MUFAs required for membrane biogenesis to support cell proliferation (1–6, 31). Accordingly, we have previously shown that FAS and SCD1 expression are also greatly increased in primary human T-cells following the induction of cell proliferation, suggesting that this is a common phenomenon associated with cell proliferation (9). Despite significant evidence for enhanced de novo SFA and MUFA biosynthesis in proliferating cells such as carcinomas (1–6, 31, 32), there is little information on potential changes in PUFA uptake and metabolism once human T-lymphocytes enter the cell cycle (33) and no information on any changes in the expression of enzymes that catalyze these processes. In this study using primary human T-cells, we show that the induction of cell proliferation is associated with significant changes in the cellular capacities to incorporate, elongate, and desaturate PUFAs from both the n-6 and n-3 families and that this is associated with the increased expression of ELOVL5 and Δ5-desaturase.

Based on the cellular FA composition, resting primary T-cells showed no measurable capacity to desaturate any of the exogenously provided PUFAs through the Δ6- or Δ5-desaturase-catalyzed reactions. These resting cells could elongate 18:3n-6 and its homologue 18:4n-3, which are the products of the Δ6-desaturase-catalyzed reaction (Fig. 1), suggesting that FAs with a double bond at the Δ6 position are the preferred 18-carbon substrates for the elongases expressed in these cells. This pattern of 18-carbon PUFA elongation suggests that resting cells may express ELOVL5, which, when expressed in yeast, catalyzes the elongation of 18:3n-6 and 18:4n-3 but is less active with 18:3n-3 as a substrate (34). This was confirmed in Western blot experiments in which these cells expressed measureable ELOVL5 but not ELOVL2, which has also been suggested to elongate 18-carbon PUFAs. However, human ELOVL5 also utilizes 20:5n-3 very efficiently in yeast (34), but there was no measurable evidence that resting T-cells could elongate or desaturate the exogenously provided 20-carbon FAs EPA and AA. These observations suggest that the substrate preference of ELOVL isotypes observed in forced expression experiments does not necessarily reflect the actual capacity of cells to elongate FAs.

The induction of cell proliferation in human T-cells was associated with a significant increase in the cells’ capacity to take up, elongate, and desaturate PUFAs. However, there were some differences dependent on the PUFAs provided to the cells. While proliferating T-cells efficiently elongated 18:3n-3 compared with resting cells, the capacity to elongate its homologue 18:2n-6 was not as pronounced, suggesting the induction of an elongase that may show a preference for 18:3n-3. This is consistent with the elongation of 18:2n-6 and 18:3n-3 previously shown in phytohemagglutinin-activated T-cells (33). Not only was 18:3n-3 elongated in stimulated cells, but a significant fraction of the FA was further desaturated to 20:5n-3, indicating the presence of both Δ6- and Δ5-desaturase activities. The induction of elongase activity was also evident in proliferating T-cells when cells were incubated in the presence of 18:4n-3 or its homologue 18:3n-6, which were both nearly completely subjected to elongation reactions with little accumulation of the exogenously added PUFAs themselves. However, once again, only the n-3 homologue 18:4 n-3 showed any evidence of undergoing desaturation through the Δ5-desaturase-catalyzed reaction. Although multiple time points may be required to precisely measure the rate of conversion of exogenously added PUFAs, the differential FA compositions measured after a 24 h supplementation clearly demonstrated differences in the cells’ capacities to elongate/desaturate the provided PUFAs.

The changes in PUFA elongation and desaturation capacities in stimulated T-cells were accompanied by increased gene and protein expression of Δ5-desaturase as well as that of the elongase ELOVL5. Interestingly, although the gene expression of both FADS1 and FADS2 was similarly elevated, the impact on Δ6-desaturase protein content was not as great as that on the Δ5-desaturase. This suggests the regulation of the expression of these two proteins also involves mechanisms other than gene expression that are not equivalent for both proteins. This is the first report of the induction of the expression of these desaturase and elongase enzymes in association with cell proliferation and may be related to the requirement of proliferating cells to maintain a cellular PUFA content for the biogenesis of functional membranes. Knockdown experiments clearly showed that this elongase isotype was responsible for the enhanced elongation capacities. Of note, ELOVL2, which has also been suggested to play an important role in PUFA elongation based on overexpression experiments (19) and in ELOVL2^−/−^ mice (35), was not expressed in these cells. This result is consistent with the limited expression profile of ELOVL2 in most human tissues, including an apparent lack of expression in resting leukocytes (36). Comparable results were obtained in Jurkat cells that also did not express ELOVL2 and in which ELOVL5 knockdown significantly affected 18-carbon PUFA elongation. Jurkat cells are a human leukemic T-cell lymphoblast cell line that is often used to study T-cell functions. ELOVL5 knockdown experiments also significantly decreased the majority of the elongation capacity of 18-carbon PUFAs in HepG2 hepatoma cells, despite the fact that these cells also express ELOVL2. Although ELOVL5 knockdown was not complete in these experiments, these results suggest that ELOVL5 is the main if not the sole elongase responsible for 18-carbon PUFA elongation in these cell types.

With regard to the 20-carbon PUFAs EPA and AA, the induction of T-cell proliferation was associated with a newly acquired capacity to elongate these FAs, although the conversion of EPA to 22:5n-3 was more complete than that measured for the elongation of AA, again suggesting that the n-3 PUFAs undergo elongation and desaturation more readily than their n-6 counterparts. This was especially evident in experiments using deuterated substrates. The elongation of AA in activated T-cells was previously shown; however, it was not compared with resting cells, and EPA elongation was not measured (33). Once again, ELOVL5 knockdown experiments showed that this elongase isotype was also responsible for the elongation of 20-carbon PUFAs, not only in primary T-cells but also in Jurkat cells. In HepG2 cells, ELOVL5 knockdown also eliminated the majority of AA-d_8_ elongation but was less effective at eliminating EPA-d_5_ elongation, with only a 30% reduction compared with controls, suggesting that ELOVL5 is only partially responsible for the elongation of EPA in this hepatocarcinoma cell line. There was no measurable 24:4n-6 or 24:5n-3 in any of the studied cells, which is consistent with a lack of an increase in 22:5n-6 or 22:6n-3 content in supplementation experiments. Overall, these results suggest that ELOVL2 is not associated with the elongation of PUFAs in human lymphoid cells, and despite its ability to catalyze the elongation of 20-carbon PUFAs when overexpressed in yeast, these results along with the limited tissue expression profile of ELOVL2 also cast doubt on a dominant role of this enzyme in the elongation of essential PUFAs in humans.

A noteworthy observation was that the enhanced capacity to elongate and desaturate PUFAs following the induction of T-cell proliferation was not equivalent for n-3 and n-6 families. The proportion of newly incorporated n-3 PUFAs that underwent elongation or desaturation was generally greater than that measured for the n-6 PUFAs. While this could be a reflection of a preference of desaturases and elongases for the n-3 PUFAs, other factors could explain this observation. For example, the cellular content of n-6 PUFAs was greater in control nonsupplemented cells than that of n-3 PUFAs, and the uptake of exogenous n-6 PUFAs based on the increase in cellular PUFA mass after supplementation was generally greater than the uptake of n-3 PUFAs. Therefore, the actual mass of n-6 PUFAs metabolized through the pathways was greater than that of n-3 PUFAs. In addition, because PUFAs are typically associated with GPL, the measured FA composition is also likely a function of the specificities of the ACSLs and acyl-transferases that are expressed in the cells. In addition, the murine macrophage RAW.267 cell line was recently reported to accumulate mead acid (20:3n-9) following cell culture (37); however, this was not observed in the current study because mead acid accounted for less than 1% of the FAMEs prepared from all cells under the described culture conditions.

The knockdown of ELOVL5 also had a significant impact on cellular MUFA content. Significant increases were measured in 16:1n-7 content in all three cell types, indicating that ELOVL5 is associated with the elongation of this MUFA, consistent with a previous report that ELOVL5 silencing increases cellular 16:1:16:0 ratios (38). Furthermore, ELOVL5 silencing also resulted in decreases in the cellular content of longer-chain 20-, 22-, and 24-carbon n-9 MUFAs. Although ELOVL5 overexpression in COS-7 monkey kidney fibroblast cells has shown that ELOVL5 can utilize 16- and 18-carbon SFAs and MUFAs as substrates (39), this is the first report in which ELOVL5 silencing affects the content of these long-chain MUFAs. While human ELOVL2 and ELOVL5 have been mainly associated with PUFA elongation, this study clearly shows in three different cell models that ELOVL5 may play a more prominent role in long-chain MUFA metabolism than previously recognized. Accordingly, whether an impact on MUFA metabolism is associated with the role of ELOVL5 mutations in neurodegenerative disorders such as spinocerebellar ataxia 38 (40, 41) remains to be determined.

Because ELOVL5 expression was associated with the induction of cell proliferation and appeared to be the main ELOVL isotype required for PUFA and MUFA elongations in T-cells and Jurkat cells, an attempt was made to determine whether its expression was required for Jurkat cell proliferation and survival. However, no measurable effect of ELOVL5 knockdown on cell proliferation as measured by two different methods, or on apoptosis as measured by annexin V labeling, was achieved. Similarly, ELOVL5 knockdown had no measurable effect on the expression of the T-cell activation markers CD45 and CD69 in stimulated primary T-cells. Therefore, although cellular FA elongation is largely dependent on ELOVL5 expression, its role in the maintenance of cellular functional capacities remains to be determined.

As indicated above, stimulated T-cells had a much greater capacity to incorporate PUFAs than resting T-cells, and this was true for all of the tested PUFAs. On a molar basis, proliferating cells took up approximately two times more n-6 PUFAs than n-3 PUFAs, except for AA, whose incorporation was similar to that of the n-3 PUFAs. The increased uptake of PUFAs by proliferating T-cells could be due to a number of mechanisms, including the expression of ACSLs that have been shown to play a role in PUFA incorporation by trapping newly incorporated PUFAs in the cells as CoA-esters (42–44). We previously showed that the induction of T-cell proliferation was associated with increased ACSL activity and mRNA expression of a number of ACSL isotypes compared with resting T-cells (9). Thus, the significant increase of the ACSL4 protein expression may explain this increased cellular uptake, although future experiments will be required to determine the extent to which these enzymes may be involved in the enhanced cellular PUFA uptake.

In conclusion, significant changes in the capacities to incorporate and metabolize PUFAs occur when T-cells are induced to proliferate, and these changes are accompanied by the enhanced expression of several genes and enzymes associated with these processes. These observations along with our previous findings that de novo FA biosynthesis is enhanced and that the highly unsaturated AA undergoes significant remodeling between GPL species (9) indicate that several important changes in cellular FA and GPL remodeling accompany entry into the cell cycle. Future studies will evaluate to what extent these changes in lipid handling may be required for continued progression in the cell cycle and for optimal functional responses.

## Supplementary Material

Supplemental Data
