# Supplementary material for: Polyunsaturated fatty acid elongation and desaturation in activated human T-cells: ELOVL5 is the key elongase
Source: J Lipid Res. 2018 Oct 6;59(12):2383–96. doi: 10.1194/jlr.M090050 (PMC6277159; doi:10.1194/jlr.M090050)
Supplement: Supplemental Data [file supp_59_12_2383__index.html]

Polyunsaturated fatty acid elongation and desaturation in activated human T cells: ELOVL5 is the key elongase — Polyunsaturated fatty acid elongation and desaturation in activated human T-cells: ELOVL5 is the key elongase — Supplemental Data 

# Polyunsaturated fatty acid elongation and desaturation in activated human T-cells: ELOVL5 is the key elongase

## Supplemental Data

- Supplemental data (.pdf, 2.4 MB) - Supplemental Figures and Tables
