# Supplementary material for: Polyunsaturated fatty acid elongation and desaturation in activated human T-cells: ELOVL5 is the key elongase
Source: J Lipid Res. 2018 Oct 6;59(12):2383–96. doi: 10.1194/jlr.M090050 (PMC6277159; doi:10.1194/jlr.M090050)
Supplement: Supplemental Data [file 10.1194_M090050_jlr.M090050-1.pdf]

Polyunsaturated fatty acid elongation and desaturation in activated human T cells: ELOVL5 is the key elongase

Philippe-Pierre Robichaud[*,1,2], Jean Eric Munganyiki[*,1], Eric Boilard[2] and Marc E. Surette[1]

[1]Department of Chemistry and Biochemistry, Université de Moncton, Moncton, NB, E1A 3E9, Canada.

[2]Department of Microbiology, Infectious Diseases and Immunology, Université Laval, Québec, QC, G1V 4G2, Canada

*co-first authors

Author correspondence: Marc E Surette, PhD

Department of Chemistry and Biochemistry

University of Moncton

Moncton, NB, E1A 3E9, Canada

Tel.: (506) 858-4293

Fax: (506) 858-4541

E-mail: marc.surette@umoncton.ca

Supplemental Figure S1. The percent distribution of n-6 and n-3 fatty acids in resting T cells, proliferating T cells and Jurkat cells following supplementation with different PUFA.

Supplemental Table S1. The mass content of fatty acids in resting T cells, proliferating T cells and Jurkat cells following supplementation with different n-6 PUFA.

Supplemental Table S2. The mass content of fatty acids in resting T cells, proliferating T cells and Jurkat cells following supplementation with different n-3 PUFA.

Supplemental Figure S2. Proliferation of Jurkat cells measured by the Click-iT® EdU Alexa Fluor® 488 and FxCycle flow cytometry cell proliferation assay following ELOVL5 knockdown.

Supplemental Figure S3. Annexin V / propidium iodide (PI) flow cytometry apoptosis assay.

Supplemental Figure S4. CD25 and CD69 expression in primary T cells.

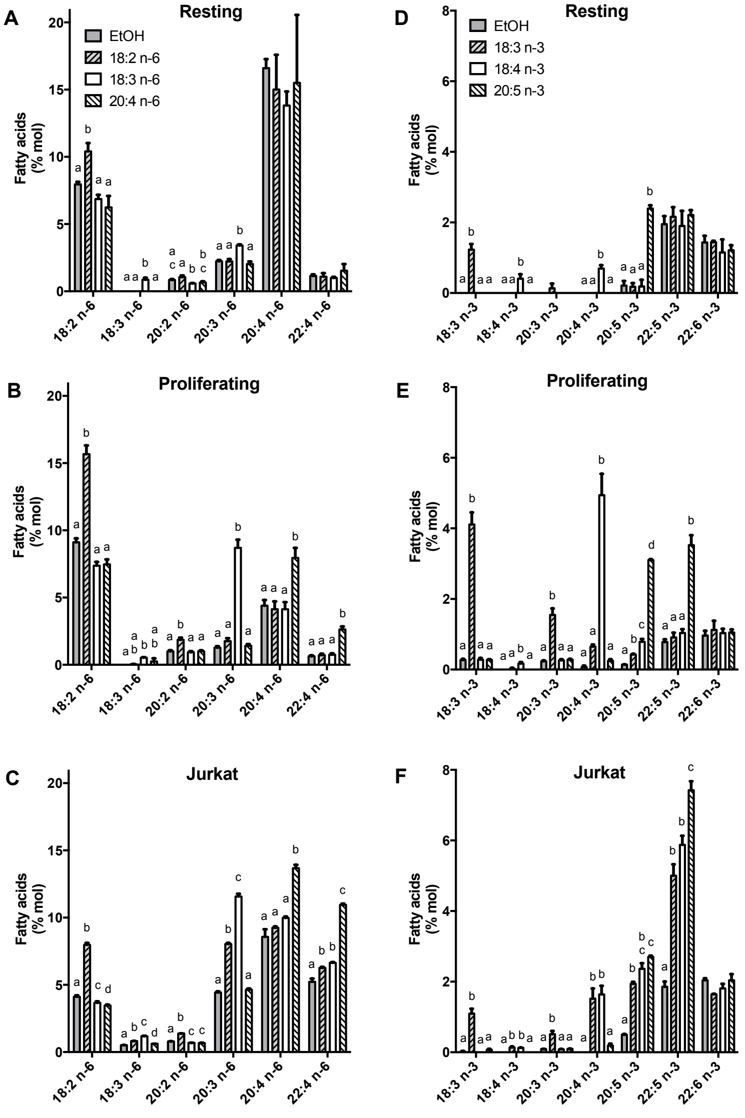

Supplemental Figure S1. The percent distribution of n-6 and n-3 fatty acids in resting T cells, proliferating T cells and Jurkat cells following supplementation with different PUFA. Resting T cells were incubated without stimulation and proliferating T cells were incubated with anti-CD3/anti-CD28 beads in the presence of 30 units/ml of IL-2 for 3 days. T cells and Jurkat cells were then incubated for 24 hours with different n-6 (**A-C**) and n-3 (**D-F**) PUFA (18:2n-6, 18:3n-6, 20:4n-6, 18:3 n-3, 18:4 n-3 or 20:5 n-3), or ethanol as control. Resting T cells **(A and D)** were incubated with 15 μM of each PUFA whereas proliferating T cells **(B and E)** and Jurkat

cells **(C and F)** were incubated with 5 μM of each PUFA. Cellular lipids were extracted, hydrolyzed and transmethylated. Individual FA were measured by GC-FID. The results are means +/- SEM of 3 (with n-3 PUFA) or 4 (with n-6 PUFA) independent experiments. Each independent experiment was conducted with cells obtained from a different subject. Values for each measured FA that do not have a common superscript are significantly different $p < 0.05$ as determined by oneway ANOVA with repeated measures and using Tukey's post hoc test.

Supplemental Table S1. The mass content of fatty acids in resting T cells, proliferating T cells and Jurkat cells following supplementation with different n-6 PUFA. Resting T cells incubated without stimulation and proliferating T cells incubated with anti-CD3/anti-CD28 beads in the presence of 30 units/ml of IL-2 for 3 days. T cells and Jurkat cells were then incubated for 24 hours with different n-6 PUFA (18:2n-6, 18:3n-6, 20:4n-6) or ethanol as control. Resting T cells were incubated with 15 μM of each FA whereas proliferating T cells and Jurkat cells were incubated with 5 μM of each PUFA. Cellular lipids were extracted, hydrolyzed and transmethylated. Individual FA were measured by GC-FID. The results are means +/- SEM of 4 independent experiments. Each independent experiment was conducted with cells obtained from a different subject. T cells were obtained from 2 males and 2 females. *Different from ethanol control ($p < 0.05$) as determined by oneway ANOVA with repeated measures and using Dunnett's post hoc test.

Supplemental Table S1

Resting	ETOH		18:2 n-6		18:3 n-6		AA	
	nM/10^8 cellules	SEM	nM/10^8 cellules	SEM	nM/10^8 cellules	SEM	nM/10^8 cellules	SEM
14:0	2.91	0.59	3.17	0.29	1.94	0.80	1.66	0.71
16:0	86.96	5.30	99.35	11.88	90.06	12.73	72.83	14.42
16:1	2.42	0.30	1.98	0.21	2.23	0.61	1.22	0.46
18:0	118.35	9.97	142.37	4.92	110.50	12.35	114.41	21.70
18:1 n-9	29.23	3.30	30.19	2.35	22.32	1.93	22.94	5.98
18:1 n-7	12.89	1.51	12.34	1.02	9.43	0.59	8.81	2.32
18:2	31.12	3.40	46.88	4.99 *	23.32	2.41	21.75	6.22
18:3 n-6	0.00	0.00	0.00	0.00	2.84	0.44 *	0.00	0.00
18:3 n-3	0.00	0.00	0.00	0.00	0.00	0.00	0.00	0.00
20:0	1.37	0.14	1.43	0.33	1.00	0.20	1.10	0.49
18:4 n-3	0.00	0.00	0.00	0.00	0.00	0.00	0.00	0.00
20:1	1.66	0.28	1.20	0.20	0.60	0.21 *	1.12	0.26
20:2 n-6	3.29	0.42	4.75	0.71	2.02	0.32 *	2.16	0.48 *
20:3 n-6	8.67	0.79	10.03	1.24	11.56	1.07	6.77	1.48
20:4 n-6	65.44	8.79	68.71	15.55	46.52	4.38	57.75	22.00
20:3 n-3	0.00	0.00	0.00	0.00	0.00	0.00	0.00	0.00
22:0	0.35	0.21	0.56	0.33	0.32	0.20	0.39	0.23
20:4 n-3	1.68	1.68	0.00	0.00	0.00	0.00	0.00	0.00
22:1	2.76	1.29	1.99	0.54	1.60	0.71	2.32	0.56
20:5 n-3	1.17	0.50	0.26	0.26	0.29	0.17	0.00	0.00 *
22:4	4.56	0.92	5.02	1.52	3.39	0.49	5.52	2.05
24:0	0.00	0.00	0.00	0.00	0.00	0.00	0.00	0.00
24:1	0.97	0.12	1.10	0.08	0.84	0.13	0.97	0.22
22:5	7.45	1.26	8.51	2.26	5.42	0.46	5.59	2.26
22:6	6.87	1.47	7.03	2.51	4.36	0.58	4.52	2.07
Total	390.10	36.51	446.85	21.32	340.56	34.92	331.83	72.89

Proliferatin	ETOH		18:2 n-6		18:3 n-6		AA	
	nM/10^8 cellules	SEM	nM/10^8 cellules	SEM	nM/10^8 cellules	SEM	nM/10^8 cellules	SEM
14:0	62.59	23.70	57.12	23.56	80.62	8.35	66.27	5.58
16:0	696.51	61.39	636.50	90.69	803.76	115.95	636.70	31.28
16:1	131.44	16.86	76.76	13.00 *	86.86	8.61	66.28	6.88 *
18:0	474.69	53.69	445.01	55.70	427.45	50.49	369.51	44.39
18:1 n-9	494.38	68.83	398.85	44.26	367.11	35.80	300.26	31.47 *
18:1 n-7	167.71	26.41	154.14	18.66	140.76	10.34	109.19	14.42 *
18:2	239.02	31.18	398.13	48.90 *	194.21	28.13	159.84	16.20
18:3 n-6	0.00	0.00	0.90	0.56	13.80	1.67 *	5.66	5.38
18:3 n-3	7.91	1.61	5.89	2.15	4.59	0.92	2.80	0.99 *
20:0	6.91	1.14	5.72	0.68	5.01	0.65	4.98	0.75
18:4 n-3	0.00	0.00	0.00	0.00	0.00	0.00	0.00	0.00
20:1	20.02	3.33	20.95	3.80	14.93	1.60	13.92	2.29
20:2 n-6	26.66	4.48	48.03	7.87 *	24.79	4.74	21.15	1.78
20:3 n-6	33.95	5.96	44.90	7.22	232.44	44.14 *	29.78	1.79
20:4 n-6	117.78	23.47	105.90	19.42	107.21	15.21	170.88	24.53
20:3 n-3	7.55	1.13	8.89	1.41	7.84	1.16	6.67	0.67
22:0	4.71	0.77	4.30	0.62	4.03	0.48	3.09	0.38
20:4 n-3	0.58	0.58	0.00	0.00	0.00	0.00	0.00	0.00
22:1	7.56	2.23	5.89	1.20	5.42	0.91	7.88	2.71
20:5 n-3	5.75	1.45	4.61	0.80	4.15	0.58	3.06	0.66 *
22:4	17.13	3.43	18.40	3.53	19.95	3.83	55.29	3.11 *
24:0	8.11	1.83	7.17	1.68	5.97	0.76	5.72	1.34
24:1	8.09	1.97	8.48	2.26	6.37	0.99	5.59	1.19
22:5	30.25	7.00	28.54	5.27	25.48	4.18	18.52	2.57
22:6	41.67	10.63	38.05	7.40	32.63	5.22	23.71	3.61
Total	2610.97	308.66	2523.10	260.47	2615.38	314.66	2138.30	174.96

Jurkat	ETOH		18:2 n-6		18:3 n-6		AA	
	nM/10^8 cellules	SEM	nM/10^8 cellules	SEM	nM/10^8 cellules	SEM	nM/10^8 cellules	SEM
14:0	60.83	4.91	64.39	5.23	47.36	1.53	58.53	5.78
16:0	879.33	51.89	909.15	54.47	728.13	15.53	907.04	51.88
16:1	79.17	5.49	80.15	4.69	65.44	1.55	79.97	6.02
18:0	463.13	25.05	449.18	20.91	350.93	9.96	495.98	53.38
18:1 n-9	715.23	44.95	689.05	42.33	529.60	11.43 *	619.44	32.99
18:1 n-7	214.09	13.71	200.62	14.27	150.30	2.26 *	176.01	9.70
18:2	146.28	11.65	323.32	15.29 *	115.70	4.65	136.79	8.04
18:3 n-6	18.18	1.59	32.93	1.82 *	36.94	2.10 *	24.01	1.29
18:3 n-3	2.49	0.46	2.74	0.26	2.50	0.36	2.92	0.21
20:0	18.09	2.78	13.44	1.38	11.90	1.43	10.87	1.03 *
18:4 n-3	0.00	0.00	0.00	0.00	0.00	0.00	0.00	0.00
20:1	46.36	2.48	44.94	2.25	29.84	0.70 *	34.25	2.11 *
20:2 n-6	28.19	2.14	56.12	3.16 *	21.44	0.57	26.36	1.19
20:3 n-6	157.93	13.75	326.46	19.37 *	366.30	14.03 *	183.34	9.59
20:4 n-6	307.40	37.42	375.35	20.46	315.51	8.61	539.43	30.56 *
20:3 n-3	3.47	0.41	3.99	0.25	3.31	0.23	3.77	0.35
22:0	14.51	2.78	11.06	1.67	10.09	1.44	8.50	0.77
20:4 n-3	0.00	0.00	0.00	0.00	0.00	0.00	0.00	0.00
22:1	7.86	0.66	7.59	0.12	5.31	0.17 *	5.92	0.26 *
20:5 n-3	14.33	1.07	12.86	0.96	10.96	0.31 *	12.12	0.59
22:4	187.03	21.06	254.41	15.40	209.55	3.08	431.78	22.25 *
24:0	29.59	3.00	30.97	1.40	25.33	1.90	34.15	1.65
24:1	7.24	0.81	7.11	0.52	5.33	0.27	5.80	0.30
22:5	83.20	11.88	90.96	6.90	67.52	1.41	79.36	4.41
22:6	67.30	12.76	73.91	6.92	55.23	2.08	62.67	3.20
Total	3551.22	249.05	4060.70	230.11	3164.50	72.34	3939.00	210.86

Supplemental Table S2. The mass content of fatty acids in resting T cells, proliferating T cells and Jurkat cells following supplementation with different n-3 PUFA. Resting T cells incubated without stimulation and proliferating T cells incubated with anti-CD3/anti-CD28 beads in the presence of 30 units/ml of IL-2 for 3 days. T cells and Jurkat cells were then incubated for 24 hours with different PUFA (18:3 n-3, 18:4 n-3 or 20:5 n-3) or ethanol as control. Resting T cells were incubated with 15 μM of each FA whereas proliferating T cells and Jurkat cells were incubated with 5 μM of each PUFA. Cellular lipids were extracted, hydrolyzed and transmethylated. Individual FA were measured by GC-FID. The results are means +/- SEM of 3 independent experiments. Each independent experiment was conducted with cells obtained from a different subject. T cells were obtained from 2 males and 1 female. *Different from ethanol control ($p < 0.05$) as determined by oneway ANOVA with repeated measures and using Dunnett's post hoc test.

Supplemental Table S2

Resting	ETOH		18:3 n-3		18:4 n-3		EPA	
	nM/10^8 cellules	SEM	nM/10^8 cellules	SEM	nM/10^8 cellules	SEM	nM/10^8 cellules	SEM
**14:0**	3.31	0.38	3.34	0.38	2.96	0.49	3.05	0.17
**16:0**	78.21	2.07	91.19	5.54	88.58	6.55	88.22	2.53
**16:1**	0.40	0.40	0.27	0.27	0.60	0.60	0.63	0.63
**18:0**	93.48	2.91	110.43	6.30	103.46	6.71	100.94	2.62
**18:1 n-9**	21.57	1.91	25.14	3.64	25.16	3.81	22.43	1.25
**18:1 n-7**	8.24	0.38	9.40	1.18	9.60	0.79	8.18	0.47
**18:2**	22.13	0.63	25.21	2.14	25.77	3.38	20.63	0.57
**18:3 n-6**	0.00	0.00	0.00	0.00	0.00	0.00	0.00	0.00
**18:3 n-3**	0.00	0.00	4.31	0.60 *	0.00	0.00	0.00	0.00
**20:0**	0.11	0.11	0.23	0.23	0.21	0.21	0.15	0.15
**18:4 n-3**	0.00	0.00	0.00	0.00	1.39	0.50 *	0.00	0.00
**20:1**	0.64	0.32	0.32	0.32	0.30	0.30	0.39	0.39
**20:2 n-6**	2.03	0.09	2.33	0.17	1.99	0.26	1.82	0.02
**20:3 n-6**	6.00	0.49	7.49	1.29	7.02	1.32	5.61	0.39
**20:4 n-6**	44.98	4.16	52.55	1.56	46.15	8.08	36.63	3.12
**20:3 n-3**	0.00	0.00	0.44	0.44	0.00	0.00	0.00	0.00
**22:0**	0.21	0.21	0.20	0.20	0.35	0.35	0.30	0.30
**20:4 n-3**	0.00	0.00	0.00	0.00	2.35	0.51 *	0.00	0.00
**22:1**	0.12	0.12	0.15	0.15	0.27	0.27	0.18	0.18
**20:5 n-3**	0.62	0.38	0.66	0.39	0.70	0.70	7.43	0.45 *
**22:4**	3.06	0.12	3.56	0.22	3.04	0.37	2.59	0.13
**24:0**	0.00	0.00	0.00	0.00	0.00	0.00	0.00	0.00
**24:1**	0.15	0.15	0.16	0.16	0.33	0.33	0.10	0.10
**22:5**	5.77	0.67	7.60	1.15	6.35	1.77	6.82	0.26
**22:6**	4.23	0.53	5.05	0.28	3.87	1.47	3.78	0.53
**Total**	295.27	1.18	350.02	21.46	329.79	30.59	309.89	7.98

Proliferatin	ETOH		18:3 n-3		18:4 n-3		EPA	
	nM/10^8 cellules	SEM	nM/10^8 cellules	SEM	nM/10^8 cellules	SEM	nM/10^8 cellules	SEM
**14:0**	69.22	16.53	72.43	4.27	82.90	11.63	75.37	13.07
**16:0**	575.60	42.17	619.04	21.55	655.92	66.52	675.59	67.03
**16:1**	106.96	15.87	83.49	9.04	96.27	22.37	88.86	20.94
**18:0**	327.21	13.50	340.87	14.74	342.12	21.61	363.28	46.05
**18:1 n-9**	311.01	21.99	305.41	7.27	303.41	37.54	308.73	45.71
**18:1 n-7**	103.09	15.11	107.80	6.32	105.24	22.07	107.72	22.59
**18:2**	148.25	18.81	156.56	17.49	159.21	17.20	158.36	14.25
**18:3 n-6**	0.00	0.00	0.00	0.00	0.00	0.00	0.00	0.00
**18:3 n-3**	4.98	0.65	82.44	9.33 *	5.81	1.10	5.80	0.86
**20:0**	2.36	1.20	2.05	1.04	1.99	1.00	2.25	1.14
**18:4 n-3**	0.00	0.00	0.61	0.49	3.48	1.02 *	0.00	0.00
**20:1**	11.85	0.84	11.83	0.97	9.89	0.89	10.79	1.31
**20:2 n-6**	14.57	2.69	17.12	3.31	16.55	3.18	17.91	2.53
**20:3 n-6**	18.27	2.60	19.54	2.27	21.76	3.21	22.45	2.90
**20:4 n-6**	55.90	4.22	59.55	6.78	63.32	4.10	62.61	3.93
**20:3 n-3**	4.31	0.74	31.18	4.76 *	5.37	0.79	5.80	0.46
**22:0**	2.18	1.09	2.02	1.03	2.49	1.26	2.41	1.29
**20:4 n-3**	1.28	0.71	12.97	0.99	101.43	16.07 *	5.36	1.29
**22:1**	2.18	1.11	2.31	1.17	2.20	1.12	2.41	1.30
**20:5 n-3**	2.57	0.21	8.56	0.15	16.45	3.12	65.02	7.43 *
**22:4**	9.22	1.46	10.94	1.73	10.43	1.15	10.27	1.08
**24:0**	4.71	0.22	4.77	0.14	4.27	0.97	4.35	1.07
**24:1**	2.80	1.40	2.79	1.40	2.60	1.38	2.81	1.47
**22:5**	14.24	2.23	18.38	3.31	21.46	3.38	74.02	9.88 *
**22:6**	17.77	3.52	22.76	6.04	21.21	3.34	21.88	2.32
**Total**	1810.50	129.08	1995.42	75.04	2055.79	204.74	2094.04	233.35

Jurkat	ETOH		18:3 n-3		18:4 n-3		EPA	
	nM/10^8 cellules	SEM	nM/10^8 cellules	SEM	nM/10^8 cellules	SEM	nM/10^8 cellules	SEM
**14:0**	25.40	5.87	34.54	4.72	34.56	7.62	38.97	16.21
**16:0**	982.87	61.89	1104.06	43.42	1002.50	138.47	1293.69	213.90
**16:1**	100.56	14.15	83.28	4.22	82.71	2.08	108.34	9.54
**18:0**	516.91	59.15	555.84	91.98	456.45	49.22	608.32	62.26
**18:1 n-9**	629.40	38.37	562.46	62.25	507.79	33.51	713.86	65.10
**18:1 n-7**	205.47	10.92	181.13	12.34	159.23	8.79	219.92	12.56
**18:2**	67.58	21.45	103.40	31.24	74.41	21.81	118.16	36.09
**18:3 n-6**	16.05	1.82	14.15	2.03	14.69	0.66	19.09	1.50
**18:3 n-3**	0.61	0.61	40.18	8.77 *	0.00	0.00	3.40	1.46
**20:0**	5.73	1.38	5.49	1.15	4.39	0.45	6.20	0.74
**18:4 n-3**	0.00	0.00	4.96	1.43 *	3.83	0.66 *	0.00	0.00
**20:1**	39.78	3.93	33.15	4.32	28.40	2.12	41.14	3.18
**20:2 n-6**	14.96	5.08	22.20	7.29	14.53	4.19	22.79	7.25
**20:3 n-6**	91.54	21.32	122.70	32.25	101.02	20.63	121.76	26.57
**20:4 n-6**	254.19	25.21	181.04	25.04	162.30	11.31	234.55	18.97
**20:3 n-3**	2.97	0.37	18.97	4.94 *	2.61	0.66	3.95	0.99
**22:0**	1.64	0.03	1.35	0.21	1.36	0.10	1.74	0.11
**20:4 n-3**	0.00	0.00	56.54	15.25 *	52.67	13.31 *	9.92	1.74
**22:1**	10.45	4.08	14.79	4.51	8.64	3.51	14.24	7.57
**20:5 n-3**	16.06	1.90	68.86	6.34 *	72.89	3.80 *	114.42	14.20 *
**22:4**	124.66	17.48	100.69	19.31	88.16	7.50	129.90	13.17
**24:0**	13.42	1.73	10.29	1.69	8.61	0.86	14.02	0.89
**24:1**	5.96	1.14	8.20	1.95	5.04	0.42	7.44	1.07
**22:5**	61.01	9.81	181.06	30.50	182.12	13.38	317.63	52.71 *
**22:6**	66.11	3.96	58.61	6.45	55.82	3.94	84.70	3.57
**Total**	3254.71	270.88	3567.93	404.97	3124.73	333.39	4248.15	568.66

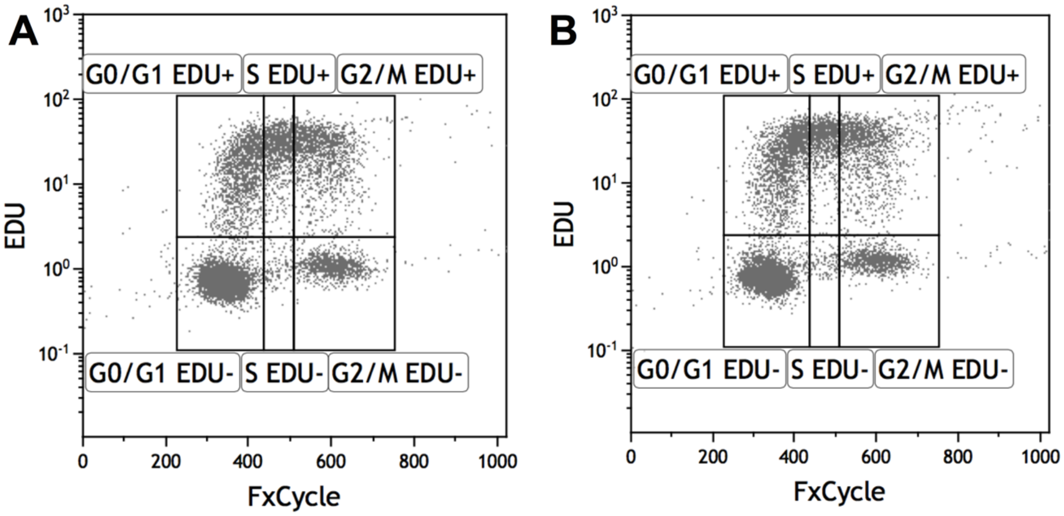

A

72h post-transfection		
Gates	Average of 3 experiments (%)	SEM
G0/G1 EDU-	43,90	2,16
G0/G1 EDU+	16,99	0,25
G2/M EDU-	10,83	0,66
G2/M EDU+	16,08	0,73
S EDU-	1,46	0,43
S EDU+	9,80	0,25

96h post-transfection		
Gates	Average of 3 experiments (%)	SEM
G0/G1 EDU-	44,45	0,79
G0/G1 EDU+	15,49	2,26
G2/M EDU-	10,92	1,06
G2/M EDU+	17,39	0,60
S EDU-	1,19	0,21
S EDU+	9,50	0,33

B

72h post-transfection		
Gates	Average of 3 experiments (%)	SEM
G0/G1 EDU-	44,57	2,67
G0/G1 EDU+	18,42	1,44
G2/M EDU-	10,29	1,29
G2/M EDU+	14,76	1,72
S EDU-	1,35	0,19
S EDU+	9,54	0,68

96h post-transfection		
Gates	Average of 3 experiments (%)	SEM
G0/G1 EDU-	46,72	0,14
G0/G1 EDU+	17,80	1,28
G2/M EDU-	9,73	0,36
G2/M EDU+	14,18	1,56
S EDU-	1,53	0,52
S EDU+	9,30	0,25

Supplemental Figure S2. Proliferation of Jurkat cells measured by the Click-iT® EdU Alexa Fluor® 488 and FxCycle flow cytometry cell proliferation assay following ELOVL5 knockdown. Cellular proliferation of Jurkat cells transfected with the non-silencing control siRNA (siRNA NS) (**A**) and the siRNA against the ELOVL5 (siRNA ELOVL5) (**B**) was analysed by flow cytometry following the manufacture's protocol. Briefly, at 72h and 96h post-transfection, Jurkat cells were incubated with 10 μM EDU for 2h at 37° C, cells were washed, fixed and permeabilized and the click-iT reaction was done to conjugate the incorporated EDU molecules to Alexa Fluor 488. Cells were then resuspended in PBS and stained with the FxCycle™ Violet Stain following the manufacture's protocol before their analysis by flow cytometry (FC500 from Beckman). The cytogram results are from one representative experiment. The tabulated results are percent of cells in each gate expressed as the means +/- SEM of 3 independent experiments.

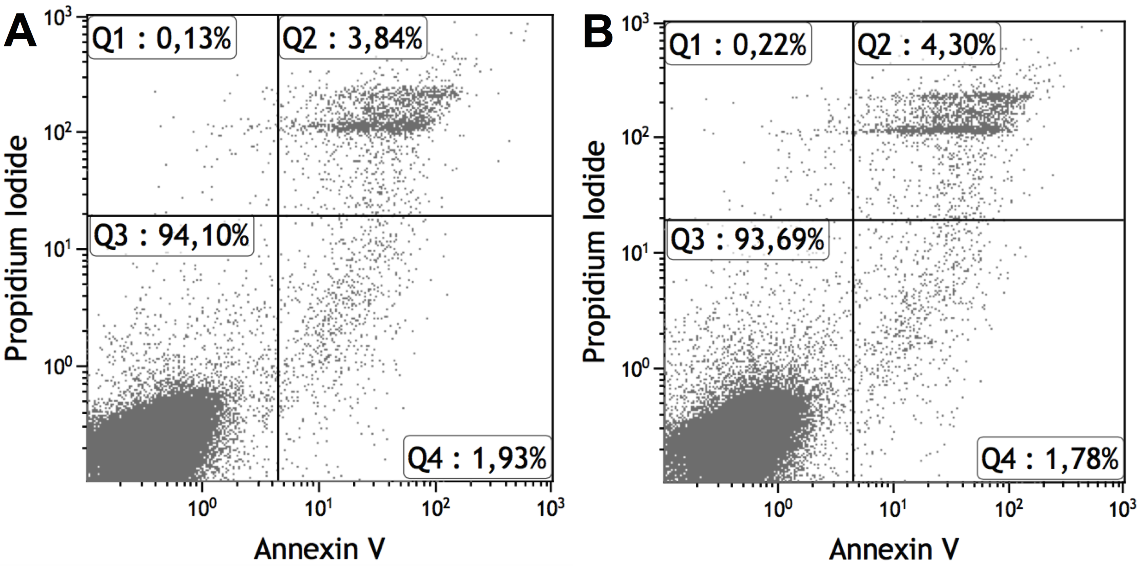

Quadrants	Average of 3 experiments (%)	SEM
Q1	0,13	0,05
Q2	4,36	0,26
Q3	92,53	0,96
Q4	2,98	0,76

Quadrants	Average of 3 experiments (%)	SEM
Q1	0,14	0,04
Q2	4,71	0,29
Q3	93,04	0,50
Q4	2,09	0,25

Supplemental Figure S3. Annexin V / propidium iodide (PI) flow cytometry apoptosis assay. Jurkat cells transfected with the non-silencing control siRNA (siRNA NS) (**A**) and the siRNA against the ELOVL5 (siRNA ELOVL5) (**B**) were incubated for 72h and were then stained with annexin V and with propidium iodide following the BioLegend protocol and analysed by flow cytometry. The cytograms are representative of 3 independent experiments. The tabulated results are percent of cells in each gate expressed as means +/- SEM of 3 independent experiments.

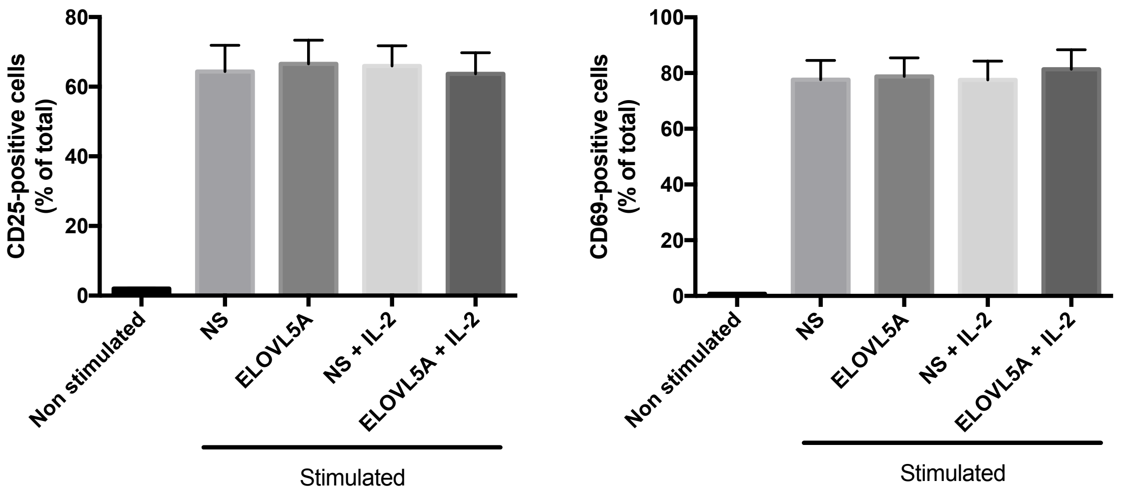

Supplemental Figure S4. CD25 and CD69 expression in primary T cells. T cells were stimulated with anti-CD3/CD28 in the presence or absence of IL-2 for 18h and were then transfected with the non-silencing control siRNA (NS) and the siRNA against the ELOVL5 (ELOVL5) as described in the methods. Cell were then incubated for 48h and were stained with PC7-conjugated anti-CD45 or PE-conjugated anti-CD69 and analysed by flow cytometry. Non-stimulated T cells are presented as a negative control. The data are the means +/- SEM of 3 independent experiments. There were no significant differences amongst stimulated cells as determined by oneway ANOVA using Tukey's post hoc test with alpha set at 0.05.
